# A Radiomics Signature-Based Nomogram to Predict the Progression-Free Survival of Patients With Hepatocellular Carcinoma After Transcatheter Arterial Chemoembolization Plus Radiofrequency Ablation

**DOI:** 10.3389/fmolb.2021.662366

**Published:** 2021-08-31

**Authors:** Shiji Fang, Linqiang Lai, Jinyu Zhu, Liyun Zheng, Yuanyuan Xu, Weiqian Chen, Fazong Wu, Xulu Wu, Minjiang Chen, Qiaoyou Weng, Jiansong Ji, Zhongwei Zhao, Jianfei Tu

**Affiliations:** ^1^ Key Laboratory of Imaging Diagnosis and Minimally Invasive Intervention, Fifth Affiliated Hospital, Wenzhou Medical University, Lishui, China; ^2^ Department of Intervention, Lishui Hospital of Zhejiang University, Lishui, China; ^3^ Department of Radiology, Lishui Hospital of Zhejiang University, Lishui, China; ^4^ Department of Pathology, Lishui Hospital of Zhejiang University, Lishui, China; ^5^ Intervention of Department, Key Laboratory of Imaging Diagnosis and Minimally Invasive Intervention, Fifth Affiliated Hospital, Wenzhou Medical University, Lishui, China

**Keywords:** nomogram, prediction, progression-free survival, transarterial chemoembolization plus radiofrequency ablation, hepatocellular carcinoma

## Abstract

**Objective:** The study aims to establish an magnetic resonance imaging radiomics signature-based nomogram for predicting the progression-free survival of intermediate and advanced hepatocellular carcinoma (HCC) patients treated with transcatheter arterial chemoembolization (TACE) plus radiofrequency ablation

**Materials and Methods:** A total of 113 intermediate and advanced HCC patients treated with TACE and RFA were eligible for this study. Patients were classified into a training cohort (*n* = 78 cases) and a validation cohort (*n* = 35 cases). Radiomics features were extracted from contrast-enhanced T1W images by analysis kit software. Dimension reduction was conducted to select optimal features using the least absolute shrinkage and selection operator (LASSO). A rad-score was calculated and used to classify the patients into high-risk and low-risk groups and further integrated into multivariate Cox analysis. Two prediction models based on radiomics signature combined with or without clinical factors and a clinical model based on clinical factors were developed. A nomogram comcined radiomics signature and clinical factors were established and the concordance index (C-index) was used for measuring discrimination ability of the model, calibration curve was used for measuring calibration ability, and decision curve and clinical impact curve are used for measuring clinical utility.

**Results:** Eight radiomics features were selected by LASSO, and the cut-off of the Rad-score was 1.62. The C-index of the radiomics signature for PFS was 0.646 (95%: 0.582–0.71) in the training cohort and 0.669 (95% CI:0.572–0.766) in validation cohort. The median PFS of the low-risk group [30.4 (95% CI: 19.41–41.38)] months was higher than that of the high-risk group [8.1 (95% CI: 4.41–11.79)] months in the training cohort (log rank test, z = 16.58, *p* < 0.001) and was verified in the validation cohort. Multivariate Cox analysis showed that BCLC stage [hazard ratio (HR): 2.52, 95% CI: 1.42–4.47, *p* = 0.002], AFP level (HR: 2.01, 95% CI: 1.01–3.99 *p* = 0.046), time interval (HR: 0.48, 95% CI: 0.26–0.87, *p* = 0.016) and radiomics signature (HR 2.98, 95% CI: 1.60–5.51, *p* = 0.001) were independent prognostic factors of PFS in the training cohort. The C-index of the combined model in the training cohort was higher than that of clinical model for PFS prediction [0.722 (95% CI: 0.657–0.786) vs. 0.669 (95% CI: 0.657–0.786), *p*＜0.001]. Similarly, The C-index of the combined model in the validation cohort, was higher than that of clinical model [0.821 (95% CI: 0.726–0.915) vs. 0.76 (95% CI: 0.667–0.851), *p* = 0.004]. The calibration curve, decision curve and clinical impact curve showed that the nomogram can be used to accurately predict the PFS of patients.

**Conclusion:** The radiomics signature was a prognostic risk factor, and a nomogram combined radiomics and clinical factors acts as a new strategy for predicted the PFS of intermediate and advanced HCC treated with TACE plus RFA.

## Introduction

Primary liver cancer (PLC) is the sixth most common malignant cancer and the fourth leading cause of cancer-related death worldwide (Bray et al., 2018). A total of 854,000 new cases are diagnosed every year, and almost 50% of PLCs come from China, which places a heavy burden on Chinese health care (Chen et al., 2018). Hepatocellular carcinoma (HCC) accounts for 75–85% of PLC, which cause a heavy burden of death in China. Approximately 70% of patients with HCC are diagnosed with advanced HCC and thus have missed the best opportunity for surgery (European Association for the Study of the Liver, 2018). The 5 years recurrence rate of HCC is as high as 70%. Local interventional strategies such as transarterial arterial chemoembolization (TACE), radiofrequency ablation (RFA) and radioactive particle implantation are widely used in the treatment of advanced HCC (Makary et al., 2020). The combination of TACE and RFA therapy has a synergistic cytotoxic effect on HCC and results in better local tumour control and longer survival than TACE or RFA alone (Peng et al., 2013; Shimose et al., 2019; Chang et al., 2020; Yuan et al., 2021).

TACE pretreatment reduces or eliminates the “heat sink” caused by blood flow during the RFA procedure and clearly shows lesion contours on CT because of lipiodol deposition. These factors may broaden the extent of coagulation necrosis and ultimately result in better outcomes (Iezzi et al., 2016). In addition, the tissue oedema induced by TACE pretreatment enlarges the necrotic area produced by RFA, thereby reducing local recurrence and increasing the safety of the ablation procedure. Use of the TACE procedure after RFA was shown to have a therapeutic effect on the sublethal heating zone of the tumour (Iezzi et al., 2016), which was the main source of local recurrence (Su et al., 2018; Tan et al., 2019). However, the 1-, 3- and 5-years local tumor progression rates reached 9, 40, 55%, respectively (J. H. Kim et al., 2011). What’s more, as a malignant tumor with obvious heterogeneity, the therapeutic effects of TACE combined with RFA were significantly various. Therefore, development of an accurate prediction model for the progression-free survival (PFS) of these patients is urgently needed.

Efforts have been made to select appropriate candidates for TACE and RFA treatment based on the Barcelona Clinic Liver Cancer (BCLC) staging system and on histopathologic grade, AFP and performance status as well as vascular invasion (Makary et al., 2020). In addition, many risk assessment models have been established to predict recurrence after TACE or RFA treatment (Canale et al., 2018; Wang et al., 2019; Han et al., 2020; Kobe et al., 2020). However, candidate patient selection and prognostic assessment models for patients with intermediate and advanced HCC who have been treated with TACE plus RFA have rarely been reported. Thus, an accurate prognostic prediction model is needed for HCC patients after TACE combined with RFA treatment.

Radiomics is a field that involves tumour segmentation, feature extraction and radiomics model building using the high-throughput mining of quantitative image features extracted from images such as magnetic resonance imaging (MRI) and computed tomography (CT) as well as positron emission computed tomography (Lambin et al., 2017; Mayerhoefer et al., 2020). Radiomics approaches have been identified as efficient strategies for predicting the risk of recurrence and survival in HCC patients (Guo et al., 2019; Hui et al., 2018; Kim et al., 2018; Peng et al., 2018). Radiomics analysis has been reported to have specific advantages. It can provide additional, reliable information that radiologists cannot obtain through analysis with the naked eye and can avoid some mistakes made by radiologists who draw conclusions based on their own personal experience. A previous study suggested that a radiomics nomogram served as a non-invasive preoperative prediction method and exhibited favourable predictive accuracy for microvascular invasion in patients with HBV-related HCC (J. Peng et al., 2018). A study by Kim et al. (2018) indicated that the combination of radiomics signature and clinical factors could accurately predict the survival of TACE-treated HCC patients. Our previous study also indicated that the radiomics and clinical indicator-based predictive nomogram can well predict tumor response in intermediate-advanced HCC (Kong et al., 2021).

This study aimed to develop and verify a novel radiomics model including a radiomics signature and clinical risk factors that can be used to predict the PFS of intermediate and advanced HCC patients treated with a combination of TACE and RFA.

## Materials and Methods

### Study Design and Patient Population

**FIGURE 1:**
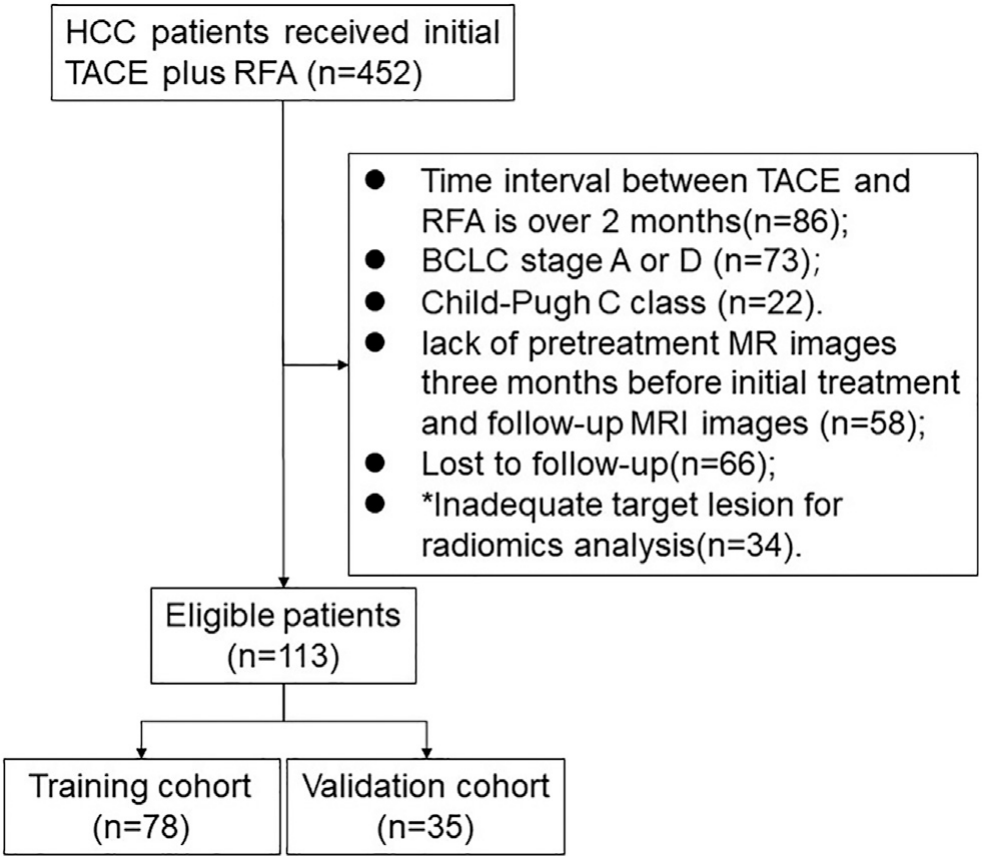
Flowchart of patient selection. * Note: The inadequate target lesion for radiomics analysis is defined as the diameter of lesion less than 1 cm.

This retrospective study was approved by the institutional ethics committee. A total of 452 HCC patients who received initial TACE plus RFA from January 2010 to December 2018 were initially included. The exclusion scheme is shown in Figure 1. The exclusion criteria were as follows: (I) time interval between TACE and RFA of more than 2 months (*n* = 86) (II) BCLC stage A or D (*n* = 73) (III) Child-Pugh class C (22) (IV) lack of available pretreatment MR images 3 months before initial treatment and of follow-up MRI images (*n* = 58) (V) lost to follow-up (*n* = 66) (VI) target lesion inadequate for radiomics analysis (*n* = 34). Finally, 113 patients were analysed in this study.

#### MR Scanning

All patients were scanned using a 3.0 T MR scanner (Philips Medical Systems, Eindhoven, Netherlands) before and after TACE plus RFA treatment. Dynamic contrast MRI images were obtained by injecting dimeglumine gadopentetate (Guangzhou Kangchen Pharmaceutical Co. Ltd.) at a dose of 0.1 mmol per kilogram body weight. The scanning parameters were as follows: 1) spectral presaturation with inversion recovery T2-weighted sequence (3,000/200 ms repetition time/echo time (TE), 7 mm slice thickness, 1 mm interslice gap, 200 × 195 matrix size); 2) breath-hold unenhanced and contrast-enhanced mdIXON-T1WI (water) sequence (3.6/1.31/2.2 ms repetition time/TE1/TE2; 400–314 mm view field, 5 mm slice thickness; −2.5 mm slice gap; 224 × 166 matrix size) for four dynamic phases: hepatic arterial phase (15 s), portal venous phase (50 s), substantial period phase (90 s) and delayed phase (180 s); and 3) breath-hold diffusion-weighted echoplanar sequence (2,500/64 ms repetition time/TE; 400–343 mm field view; 7 mm section thickness; 1 mm intersection gap; 116 × 97 matrix size; b value = 0 and 800 s/mm^2^). MRI images were exported in DICOM format.

#### TACE Procedure

All 113 eligible patients were successfully treated with TACE. All TACE procedures were performed by interventional radiologists with no less than 10 years of clinical experience according to the practice guidelines. Briefly, the femoral artery was punctured at the groin area in the region showing the strongest pulsation using the Seldinger technique. A 2.7-Fr microcatheter (Progreat; Terumo, Japan) was carefully inserted into the blood supply artery of the lesion, and the chemotherapeutic and embolic agents were injected through the microcatheter under DSA. Angiography was performed before the end of embolization to confirm satisfactory embolization of the feeding artery.

#### RFA Procedure

Percutaneous RFA was described in our previous study (W. Liu et al., 2020). Briefly, under the guidance of CT, the appropriate needle path and depth were calculated. After local anaesthesia at the puncture site and path using 1–2% lidocaine (5–15 ml), an RF electrode was inserted into the target lesion. The RF current was emitted for 10–15 min by an RF generator depending on the increased impedance. Overlap ablations were performed within the effective ablation range covering 0.5–1.0 cm outside the edge of the target lesion. Needle path ablation was performed to avoid bleeding and implant metastasis.

#### Follow-Up

PFS was the end point of this study. PFS was defined as the time from the date of TACE until the date of relapse (relapse refers to intrahepatic recurrence or extrahepatic metastasis) or until the date on which the patient was last known to be free of relapse. The first outpatient clinic visits were conducted within 4–6 weeks after TACE plus RFA treatments and every 2–3 months thereafter MRI images were obtained and used to track tumour progression. Serum AFP levels, liver function and blood cell levels were also tested.

#### Features Extraction and Selection

Two experienced radiologists with more than 10 years of experience in clinical HCC diagnosis independently and semi-automatically conducted delineation of the volume of interest (VOI) using ITK-SNAP (www.itksnap.org). All preoperative contrast-enhanded T1-weighted images were then imported into Artificial Intelligence Kit software (A.K., GE Healthcare, China) to obtain quantitative VOI information. A total of 396 radiomics features divided into five categories, including histogram, form factors, grey-level co-occurrence matrix (GLCM), grey-level size zone matrix (GLSZM) and run-length matrix (RLM), were extracted.

#### Intra-Observer and Interobserver Reproducibility

Intra- and interclass correlation coefficients (ICCs) were applied to evaluate the intra- and inter-observer agreement of VOI segmentation and radiomics feature extraction. The two readers obtained the radiomics features twice with at least a 1-month interval between the two readings, and the inter-observer agreement and intraclass correlation were computed. An ICC value greater than 0.75 was considered to indicate good agreement.

#### Radiomics Model Building and Accuracy Evaluation

The features were then standardized and normalized by the Z score method, and abnormal values were replaced with the median of the parameter. Before dimension reduction, all patients were randomly divided into a training cohort (*n* = 78 cases) and a validation cohort (*n* = 35 cases) for radiomics model building and verification, respectively. The least absolute shrinkage and selection operator (LASSO) logistic regression algorithm was further conducted for feature selection in the training cohort, and a rad-score was calculated for each patient based on the selected radiomics features in both the training and validation cohorts using Cox-regression. The predictive accuracy of the radiomics signature was evaluated based on the area under the curve (AUC) of the receiver operator characteristic (ROC) curve in the training cohort and verified in the validation cohort. An optimal cut-off value of the rad-score was calculated based on Youden index and used to classify the patients into a high-risk group and a low-risk group for recurrence-free survival analysis. The PFS in the two groups was analysed by the Kaplan-Meier method, and the difference was calculated using the log-rank test.

#### Clinical Model and Combined Model Building

Univariate Cox proportional hazard regression analysis was applied to select the clinical characteristics with *p*-values < 0.05. The potential clinical characteristics related to recurrence-free survival from the univariate analysis were identified by multivariate Cox proportional hazard analysis. The clinical model was established using the characteristics with *p*-values < 0.05 in multivariate analysis. Again, the radiomics signature were integrated into the clinical model to develop a combined model, which include clinical prognostic factors and radiomics signature. The AUC of each model was calculated to quantify predictive accuracy in both the training cohort and the validation cohort. Finally, a nomogram using radiomics signature and clinical factors from clinical model to generate a probability for individuals was built and verified by the concordance index (C-index), calibration curve, decision curve and clinical impact curve.

#### Statistical Analysis

All statistical analyses were performed using SPSS software (SPSS version 22.0) and R software (R version 4.0.3). *p*-values < 0.05 were defined as statistically significant.

Categorical variables are shown as frequencies, and continuous variables are presented as mean and standard deviation or as median and 95% confidence interval (CI) values. Categorical variables were compared using the χ^2^ test and Fisher’s exact test. Student’s t-test was used to determine the significant difference between the training and validation groups for normally distributed data; otherwise, the Mann-Whitney test was used. Feature selection was performed using the LASSO logistic regression model, which was conducted by 10-fold cross-validation. Univariate analysis using Cox’s proportional hazards regression model was applied to select the variables for multivariate analysis. The concordance index (C-index) was used to evaluate the discrimination of the radiomics, clinical and combined prediction models. The agreement between predictions from the model and observed outcomes were measured by calibration curve, assessed by calculating the Hosmer–Lemeshow goodness-of-fit test. The clinical usefulness of the prediction models was estimated based on decision curves and clinical impact curve.

## Results

### Patient Demographics and Clinical Characteristics

A total of 113 patients were eligible for this study; among these, 80 patients (70.79%) had confirmed tumour recurrence according to the clinical final observer outcome. The median PFS was 13.20 months, and the 95% CI was 9.78–16.61. The overall 1-, 2-, and 3-years cumulative PFS rates of the patients were 57.54, 31.34, and 20.37%, respectively. There were no significant differences in sex, age, HBsAg, cirrhosis, BCLC stage, Child-Pugh classification, AFP level, tumour diameter, node number, metastasis, portal vein thrombosis, TACE-RFA procedures and time interval between TACE and RFA treatment in the training and validation cohorts (Table 1).

**TABLE 1:** Characteristic of patients in the training and validation cohorts.

Characteristic	Training cohort (*n* = 78)	Validation cohort (*n* = 35)	Test value	*p* value
Gender	—	—	1.85	0.17
Male	73(93.58%)	30(85.71%)	—	—
Female	5(6.42%)	5(14.29%)	—	—
Age	—	—	1.18	0.28
<55 y	29 (37.66%)	17 (48.57%)	—	—
≥55 y	33 (62.34%)	18 (51.43%)	—	—
Child-Pugh class	—	—	1.59	0.21
A	56(71.79%)	29(82.86%)	—	—
B	22(28.21%)	6(17.14%)	—	—
Cirrhosis	—	—	1.06	0.30
Yes	70(89.74%)	29(82.86%)	—	—
No	8(10.26%)	6(17.14%)	—	—
HBsAg	—	—	0.42	0.52
Positive	72(93.31%)	31(88.57%)	—	—
Negative	6(6.69%)	4(11.43%)	—	—
BCLC stage	—	—	1.30	0.25
B	49(47.43%)	18(54.28%)	—	—
C	29(52.57%)	17(45.71%)	—	—
Tumor size (cm)	5.99 ± 2.10 cm	5.70 ± 1.89 cm	0.51	0.48
≤5 cm	38(48.71%)	20(57.14%)	0.69	0.41
>5 cm	40(51.29%)	15(42.86%)	—	—
Node	—	—	0.73	0.39
single	19(24.36%)	6(17.14%)	—	—
multiple	59(75.64%)	29(82.86%)	—	—
AFP	—	—	2.86	0.09
<200 ng/ml	19(24.36%)	14(40.00%)	—	—
≥200 ng/ml	59(75.64%)	21(60.00%)	—	—
Metastasis	—	—	0.23	0.63
Yes	14(17.94%)	5(14.28%)	—	—
No	64(82.06%)	30(85.72%)	—	—
PVTT	—	—	0.08	0.78
Yes	16(20.51%)	8(22.86%)	—	—
No	62(79.49%)	27(77.14%)	—	—
TACE-RFA procedures	1.76 ± 1.00 times	1.97 ± 1.01 times	0.09	0.30
one time	41(52.56%)	14(40.00%)	2.17	0.15
No less than two times	37(47.43%)	21(60.00%)	—	—
Time interval	—	—	2.13	0.14
<14 d	33(42.31%)	20(57.14%)	—	—
≥14 d	45(57.69%)	15(42.83%)	—	—
PFS	12.57(9.08∼16.05)	17.73(9.70～25.77)	0.18	0.65

Abbreviations: HBsAg, hepatitis B surface antigen; BCLC stage, Barcelona Clinic Liver Cancer; AFP, Alpha-fetoprotein; PVTT, Portal vein tumor thrombus; PFS, progression free survival.

#### Radiomics Feature Extraction and Radiomics Model Building

**FIGURE 2:**
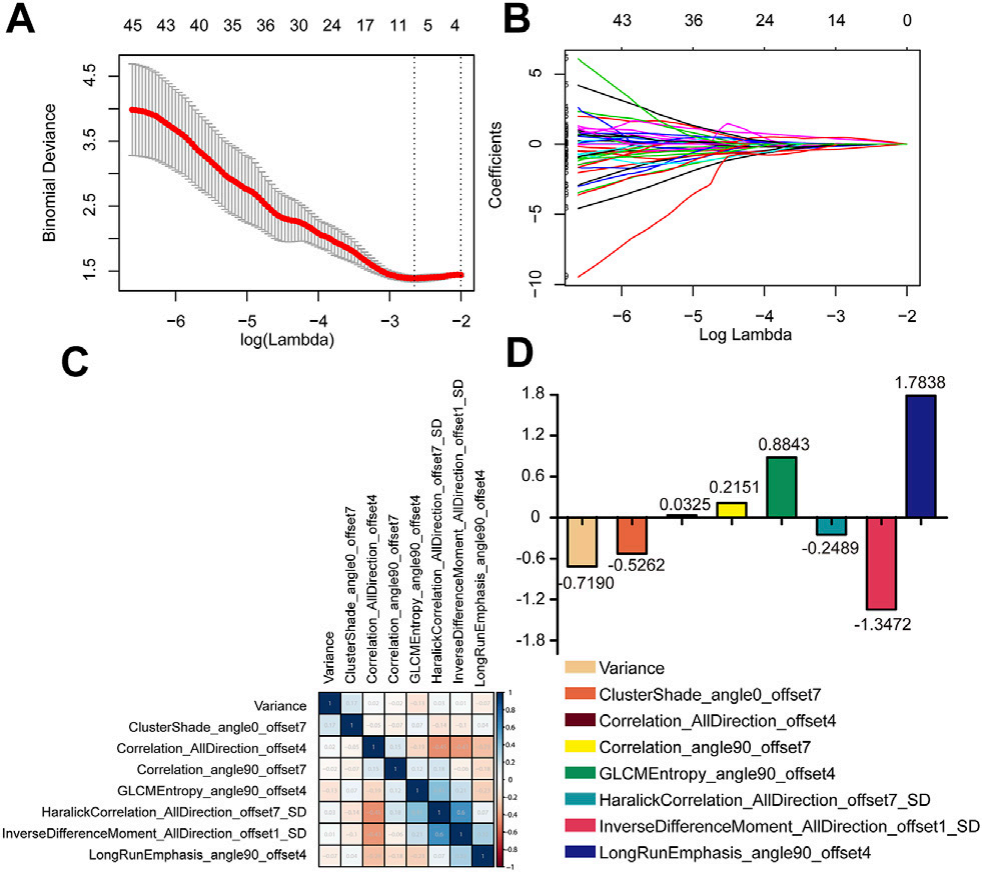
Radiomics feature selection in LASSO regression model. **(A)** The minimum criteria for 10-fold cross-validation was used to select optimal features. **(B)** LASSO coefficient analysis of 396 radiomics features. **(C,D)** The eight radiomics features were selected after dimension reduction.

Satisfactory inter- and intra-observer reproducibility of feature extraction was achieved. The intra-observer ICC calculated based on two measurements ranged from 0.81 to 0.93, and the interobserver agreement between the two readers ranged from 0.79 to 0.90. The extracted features are shown in Figure 2. Eight radiomics features were selected by the LASSO algorithm. A radiomics signature based on the eight radiomics features was built, and the formula for calculating the Rad-score for each patient was as follows: Rad-score = 0.1889–0.7190×Variance–0.5262×ClusterShade_angle0_offset7+0.0325×Correlation_AllDirection_offset4+0.2151 ×Correlation_angle90_offset7 + 0.8843×GLCMEntropy_angle90_offset4–0.2489×HaralickCorrelation_AllDirection_offset7_SD-1.3472×InverseDifferenceMoment_AllDirection_offset1_SD + 1.7838×LongRunEmphasis_angle90_offset4. The cut-off value of the rad-score was 1.62, and this value was used to classify the patients into high-risk and low-risk groups.

#### Performance of the Radiomics Model

**FIGURE 3:**
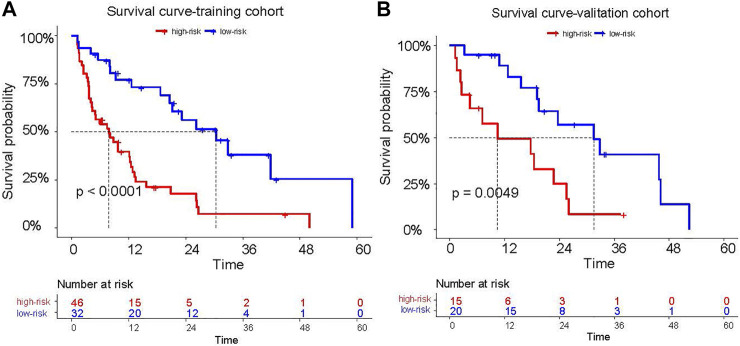
The PFS of patients who were classified into high risk and low risk patients according to the cutoff of the Rad-score **(A,B)**.

He C-index of the radiomics signature for PFS was 0.646(95%: 0.582–0.71) in the training cohort and 0.669(95% CI: 0.572–0.766) in validation cohort. In the training cohort, the median PFS was 30.4 (95% CI: 19.41–41.38) months in the low-risk group, while the median PFS was 8.1 (95% CI: 4.41–11.79) months in the high-risk group. Compared with the high-risk group, the PFS of the low-risk group was significantly longer (log rank test, z = 16.58, *p* < 0.0001, Figure 3A). In the validation cohort, the median PFS was 31.50 (95% CI: 16.99–46.00) months in the low-risk group, while the median PFS was 10.43 (95% CI: 3.07–27.62) months in the high-risk group. Compared with the high-risk group, the PFS of the low-risk group was significantly longer (log rank test, z = 7.90, *p* = 0.0049, Figure 3B).

#### Univariate and Multivariate Analyses for Prognostic Factors of PFS

Univariate Cox regression analysis showed that among the candidate variables, five variables, including BCLC stage, tumour size (≥5 cm vs. <5 cm), AFP (≥200 ng/ml vs. <200 ng/ml), interval (<14 days vs. ≥14 days) and radiomics signature (high-risk vs. low-risk), had *p* values <0.05. Multivariate analyses suggested that BCLC stage (hazard ratio (HR): 2.52, 95% CI: 1.42–4.47, *p* = 0.002), AFP level (HR: 2.01, 95% CI: 1.01–3.99, *p* = 0.046), time interval (HR: 0.48, 95% CI: 0.26–0.87, *p* = 0.016) and radiomics signature (HR 2.98, 95% CI: 1.60–5.51, *p* = 0.001) were independent prognostic factors of PFS (Table 2).

**TABLE 2:** Univariate and Multivariate analysis of factors associated with PFS.

Characteristic	Univariate analysis				Multivariate analysis			
	HR	HR	(95% CI)	*p* value	HR	HR	(95% CI)	*p* value
Age (years) (≥55 years vs <55 years)	0.71	0.45	1.11	0.14	—	—	—	—
Gender (Male vs Female)	0.68	0.29	1.58	0.37	—	—	—	—
Child-Pugh class (B vs. A)	1.43	0.88	2.32	0.15	—	—	—	—
BCLC stage (C vs. B)	2.10	1.22	3.62	**0.007**	2.52	1.42	4.47	**0.002**
Tumor size (≥5 cm vs. <5 cm)	1.83	1.07	3.14	**0.027**	—	—	—	—
Node (single vs. multiple)	1.26	0.73	2.17	0.41	—	—	—	—
AFP (≥200 ng/ml vs. <200 ng/ml)	2.58	1.31	5.06	**0.006**	2.01	1.01	3.99	**0.046**
HBsAg status (positive vs. negative)	1.69	0.68	4.20	0.26	—	—	—	—
Cirrhosis status (yes vs. no)	1.07	0.55	2.09	0.84	—	—	—	—
Metastasis (yes vs. no)	1.37	0.77	2.42	0.28	—	—	—	—
PVTT (yes vs. no)	1.41	0.82	2.42	0.22	—	—	—	—
TACE-RFA procedures	0.81	0.626	1.04	0.10	—	—	—	—
Time interval (<14 days vs. ≥14 days)	0.48	0.27	0.84	**0.010**	0.48	0.26	0.87	**0.016**
Radionics signatures (high-risk vs. low-risk)	3.26	1.79	5.93	**0.000**	2.98	1.60	5.51	**0.001**

Abbreviations: HBsAg, hepatitis B surface antigen; BCLC stage, Barcelona Clinic Liver Cancer; AFP, Alpha-fetoprotein; PVTT, Portal vein tumor thrombus; PFS, progression free survival.

Note: The *p* value marked bold indicated statistical significance.

#### Accuracy of Radiomics Signature Combined With Clinical Factors

The C-index of the combined model in the training cohort was higher than that of clinical model [0.722 (95% CI: 0.657–0.786) vs. 0.669 (95% CI: 0.657–0.786), *p* < 0.001]. similarly, The C-index of the combined model in the validation cohort, was higher than that of clinical model [0.821 (95% CI: 0.726–0.915) vs. 0.76 (95% CI: 0.667–0.851), *p* = 0.004].

#### A Novel Nomogram for Individual PFS Prediction

**FIGURE 4:**
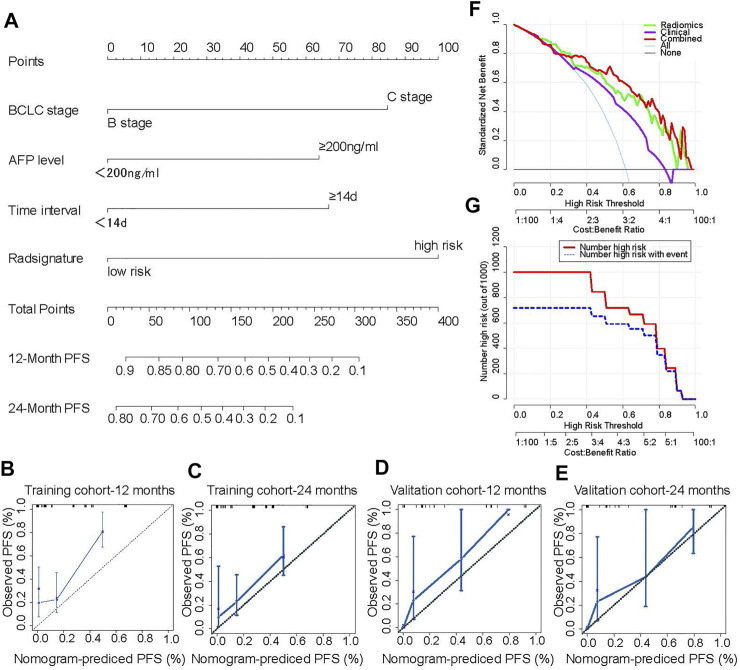
A novel nomogram for individual PFS prediction. A novel nomogram based the radiomics signatures and clinical factors that was developed in the training cohort. The probability values of PFS for each HCC patient at 12 and 24 months were showed **(A)**. The corresponding calibration curve as well as the C-index were conducted to compared the probability of PFS between the nomogram prediction and actual observation in the training and validation cohorts **(B–E)**. The net benefit for each model was calculated by using decision curve, which the abscissa indicated the threshold probability and the ordinate represented the net benefit **(F)**. The consistency between nomogram predicted recurrence and actual recurrence was compared by using clinical impact curve **(G)**. The number of patients with high-risk for recurrence who were classified as high risk by the by the model at each threshold probability was calculated (red line) and the number of patients who truly suffer from recurrence was showed at each threshold probability (blue line).

A radiomics nomogram based on the combined model including the radiomics signature and clinical characteristics was established (Figure 4A). The calibration curve showed that the nomogram-predicted PFS was consistent with the observed PFS at 12 months and at 24 months (Figures 4B–E). These results indicate that the nomogram has a good predictive effect for the PFS of patients with intermediate and advanced HCC. The decision curve results showed that at threshold probabilities of up to 18%, the nomogram with the radiomics signature alone provided a greater benefit than the “no treatment” or “all treatment” strategies. At threshold probabilities of up to 25%, the discrimination ability of the combined nomogram was better than that of the nomogram with clinical factors alone (Figure 4F). Moreover, the clinical impact curve also showed that the nomogram offered good net benefit for the identification of patients who were likely to suffer recurrence (Figure 4G).

## Discussion

Locoregional therapies, including transarterial chemoembolization (TACE) and ablations, are proposed to postpone disease progression (Palmer et al., 2020). Therapy that combines TACE and RFA has been recommended and has proven to be an effective selection for intermediate and advanced HCC (Z. W. Peng et al., 2013). However, the outcomes are not satisfactory for all individuals. Therefore, it is urgent to identify patients who may truly experience a clinical benefit. In this study, we developed a model for predicting PFS based on a radiomics signature and clinical factors. We found that the PFS of patients with a low-risk radiomics signature was higher than that of patients with a high-risk radiomics signature. The C-index of the combined model in the training cohort was higher than that of clinical model [0.722 (95% CI: 0.657–0.786) vs. 0.669 (95% CI: 0.657–0.786), *p* < 0.001]. The C-index of the combined model in the validation cohort, was higher than that of clinical model [0.821 (95% CI: 0.726–0.915) vs. 0.76 (95% CI: 0.667–0.851), *p* = 0.004]. The model combining the radiomics signature and clinical factors provided a better evaluation of PFS than the model that considered clinical factors alone.

A radiomics approach was applied to identify more heterogeneous information from images. Radiomics signature extracted from ultrasonic, CT and MRI images have been found to predict recurrence in HCC patients who receive surgery (Akai et al., 2018), liver transplantation (Guo et al., 2019) or TACE (Song et al., 2020) as well as ablation (C. Yuan et al., 2019). In this study, a total of 396 radiomics features extracted by AK software were classified into five clusters: 42 histogram-based features, nine form factor-based features, 11 GLSZM-based features, 180 RLM-based features and 154 GLCM-based features. Histograms and form factor-based features were used to describe the grey distribution and the geometric characteristics of the region of interest. The GLSZM-, RLM- and GLCM-based features reflect the spatial arrangement of the colour or intensity of lesions. Eight potential predictors were selected from the 396 features by shrinking the regression coefficients with the LASSO method. Especially, variance is a parameter of histogram and represents the average of the squared differences from the Mean of image. Cluster Shade_angle0_offset7 is a texture parameter, which groups the similar samples according to their position and, optionally, normal into clusters. Correlation_AllDirection_offset4 is the Correlation that measures the similarity of the grey levels in neighboring pixels, telling how correlated a pixel is to its neighbor over the whole image. Correlation_angle90_offset7 and Haralick Correlation_AllDirection_offset7_SD is the parameters that measure the degree of similarity of the gray level of the image in the row or column direction, representing the local grey level correlation. the greater their value, the greater the correlation. GLCM Entropy_angle90_offset4 shows the amount of information of the image that is needed for the image compression. Inverse Difference Moment_AllDirection_offset1_SD is the local homogeneity. It is high when local gray level is uniform and inverse GLCM is high. Long Run Emphasis_angle90_offset4 is a Parameter of RLM that is generated for each sample image segment as different directions. A Rad-score based on the eight features was calculated for each patient. The cut-off of the Rad-score was calculated, and the patients were classified into high-risk and low-risk groups. Previous studies demonstrated that the Rad-score could predict the recurrence of HCC patients after TACE treatment. We also found that the AUCs for PFS of the radiomics signature of patients in the training and validation cohorts were as high as 0.83 and 0.81, respectively, indicating good sensitivity and specificity for PFS prediction. In addition, the PFS of the low-risk group was higher than that of the high-risk group, further confirming that radiomics signature could be an option for PFS evaluation of HCC patients treated with TACE and RFA.

The BCLC staging system and AFP level have been proven to be efficient in predicting the survival of liver cancer. Consistent with previous studies (Ren et al., 2019; Shimose et al., 2019), we also found that BCLC stage and AFP were related to the PFS of HCC patients treated with TACE and RFA by univariate and multivariate analysis. Other clinical factors such as tumour size, the presence of multifocal lesions, HBV status and neoplasm grade have been recommended for use in evaluating the short- and long-term outcomes of liver cancer. Studies have indicated that tumour size is an independent prognostic factor for recurrence in HCC patients receiving TACE with or without RFA (Sieghart et al., 2015; H. Liu et al., 2016). In contrast, a study by Peng (Z. W. Peng et al., 2013) showed that tumour size was not associated with recurrence-free survival of HCC. In this study, we also found that tumour size was excluded from the multivariate model and was not a prognostic factor for intermediate or advanced HCC. These discrepancies may be due to selection bias.

Studies have demonstrated that TACE shows synergistic effects with RFA in improving tumour response and survival in intermediate or advanced HCC patients (Z. W. Peng et al., 2013; P. Yuan et al., 2021; W. Liu et al., 2020). However, consensus regarding the optimal time interval between TACE and RFA for balancing efficacy and safety remains unclear. A shorter time interval might increase the potential risk of liver dysfunction. Conversely, a longer time interval favours recovery of liver function but may prolong the hospital stay and might decrease the local efficacy of the combination therapy. It is suggested that the use of a time interval between 1 and 30 days could amplify the synergistic effects of the combination therapy (Choe et al., 2014; Iezzi et al., 2016). In this study, we also showed that an interval of 14 days or less was a predictive factor for PFS, consistent with the study by Peng (Z. W. Peng et al., 2013).

A nomogram was used to provide individual predictions; the accuracy of the predictions was verified by calculating the C-index and AUC, and they were analysed based on the calibration curve, the decision curve and the clinical impact curve (Guo et al., 2019; C. Yuan et al., 2019; Song et al., 2020; Zheng et al., 2020). In this study, three factors (BCLC stage, AFP and time interval) with or without a radiomics signature were used to develop clinical and combined prediction models. The AUC of the combined model was higher than that of the clinical model in both the training and the validation cohorts, indicating better performance of the combined model. Thus, a nomogram incorporating the above four factors was constructed. The nomograms in the training and validation cohorts showed favorable consistency, with C-index values of 0.722 (95% CI: 0.657–0.786) and 0.821 (95% CI: 0.726–0.915), respectively. The decision curve suggested that when the threshold probability was approximately 18%, application of the nomogram with the radiomics signature alone for PFS prediction provided a greater benefit than the non-treatment or overall-treatment strategies. The probability of PFS based on a clinical model ranges from 38 to 82%. The net benefits of the radiomics signature alone and the combined nomogram were higher than that of the clinical model alone. Moreover, the outcome was verified by a clinical impact curve.

Admittedly, this study has certain limitations. As a single-centre and retrospective study, there was inevitable selection bias. Almost half of HCC patients in this study were in BCLC stage C, which have great impact on the outcome owing to the fact that the first-line treatment for these patients should be systemic therapy rather than the combination of TACE and RFA. The PFS evaluation did not comprehensively reflected the overall biological behaviours of these patients, especially who with extrahepatic metastasis. A more accurate evaluation for these patients received TACE and RFA treatment should be further investigated in future study. Besides, the sample size was relatively small, and larger datasets and external validation are urgently needed to validate our findings. In the future, large-sample, multicentre cohorts will be examined to verify the prognostic significance of these radiomics signature.

## Conclusion

This study provides a radiomics signature-based nomogram of PFS prediction for intermediate and advanced HCC patients treated with TACE and RFA. The radiomics signature was a prognostic risk factor, and the nomogram combined radiomics and clinical factors acts as a new strategy for predicting the PFS of intermediate and advanced HCC treated with TACE plus RFA. These results could be used to facilitate HCC treatment management.

## Data Availability

The raw data supporting the conclusion of this article will be made available by the authors, without undue reservation.
